# Electronic Health Record Portal Use by Family Caregivers of Patients Undergoing Hematopoietic Cell Transplantation: United States National Survey Study

**DOI:** 10.2196/26509

**Published:** 2021-03-09

**Authors:** Vibhuti Gupta, Minakshi Raj, Flora Hoodin, Lilian Yahng, Thomas Braun, Sung Won Choi

**Affiliations:** 1 Department of Pediatrics University of Michigan Ann Arbor, MI United States; 2 Department of Kinesiology and Community Health University of Illinois at Urbana Champaign Champaign, IL United States; 3 Department of Psychology Eastern Michigan University Ypsilanti, MI United States; 4 Department of Psychiatry University of Michigan Ann Arbor, MI United States; 5 Center for Survey Research Indiana University Bloomington, IN United States; 6 Department of Biostatistics School of Public Health University of Michigan Ann Arbor, MI United States

**Keywords:** hematopoietic stem cell transplantation, caregiver, mobile apps, questionnaire, survey, app, cancer, electronic health record, EHR, online portal, transplant, stem cell, management

## Abstract

**Background:**

As family caregivers of patients undergoing hematopoietic cell transplantation have multifaceted caregiving responsibilities (such as medical, household, financial) of long duration, they also have multiple physical, social, psychological, and informational needs.

**Objective:**

This study explored the prevalence of electronic health record patient portal use by family caregivers for managing both their own and their hematopoietic cell transplantation care recipient’s health, as well as potential factors associated with portal use.

**Methods:**

An electronic caregiver health survey, first developed via cognitive interviewing methods of hematopoietic cell transplantation caregivers, was distributed nationally (in the United States) by patient advocacy organizations to family caregivers of hematopoietic cell transplantation patients. It was used to assess self-reported caregiver demographics, caregiving characteristics, depression and anxiety with the Patient Health Questionnaire–4, coping with the Brief COPE, and caregiver portal use to manage care recipient’s and their own health.

**Results:**

We found that 77% of respondents (720/937) accessed electronic health record patient portals for their care recipients, themselves, or both. Multivariate models indicated use of care recipient electronic health record portals by caregivers was more likely with young, White, married, low-income caregivers caring for a parent, residing with the care recipient, and experiencing more caregiver depression. Caregiver use of their own electronic health record portal was more likely with young, White, high-income caregivers caring for a parent and experiencing chronic medical conditions of their own. Partially due to multicollinearity, anxiety and coping did not contribute independently to this model.

**Conclusions:**

Findings from the survey could open avenues for future research into caregiver use of technology for informational support or intervention, including wearables and mobile health.

**International Registered Report Identifier (IRRID):**

RR2-10.2196/4918

## Introduction

### Caregivers of Patients Undergoing Hematopoietic Cell Transplantation

Hematopoietic cell transplantation is a high-risk but potentially curative therapy for life-threatening blood diseases [1-3]. Hematopoietic cell transplantation patients require a committed informal family caregiver or care partner (relative or friend) to provide unpaid assistance for long durations [4]. Caregivers of hematopoietic cell transplantation patients [5] perform complex medical tasks, transport and accompany patients during appointments, manage medications, monitor vital signs and fluid intake, assist with activities of daily living, and provide emotional support [6,7]. Caregivers experience immense psychological and physical risks resulting from the stresses of managing the care recipients’ as well as their own needs [8].

Caregiving demands often exceed the resources available to caregivers [7]. In particular, patients undergoing hematopoietic cell transplantation require caregiving for an extended time, and demands vary based on stage of disease at diagnosis, treatment intensity, and possible treatment complications [4]. If caregivers have to relocate with their care recipient to be close to the transplant center, financial toxicity and social isolation may further compound care demands [9]. Caregiving has also been described as a rewarding and positive experience; however, ensuring quality of life among caregivers of hematopoietic cell transplantation patients requires broad consideration of their physical, social, psychological, and spiritual demands and needs [4].

### Informational Needs of Caregivers and Patient Portal Utilization

Caregivers of hematopoietic cell transplantation patients have significant needs for information about their care recipient’s laboratory results, appointments, health conditions, or treatment regimens [4,10-12]. These data are available through electronic health record portals, a secure online website allowing patients access to their personal health information [10]. Caregivers may use their care recipient’s patient portal to help them with role demands, such as managing medications, keeping up-to-date with medical diagnoses and treatments, and communicating with health care providers [13,14]. Use of the patient portal can support caregivers in managing their own and their care recipient’s health [13,15]. However, little is known about hematopoietic cell transplantation caregivers’ uptake of their own portal use (self) and use of their care recipient’s portal. Information accessed via the patient portal can be critical for reducing caregiver role ambiguity and anxiety, increasing engagement in care, and meeting information needs among hematopoietic cell transplantation caregivers [10].

The purpose of this study was to learn more about family caregivers’ use of electronic health record patient portals. Building on inpatient and outpatient interviews, we developed a survey to be distributed nationally (in the United States) to family caregivers of hematopoietic cell transplantation patients—the National Caregiver Health Survey [3,16,17]. We drew upon a nationally representative sample to (1) characterize hematopoietic cell transplantation caregivers; (2) describe their mental health and coping behaviors; and (3) examine the relationship between caregiver characteristics, mental health and coping, and caregiver self and care recipient portal use.

## Methods

### Study

The survey is part of a larger multiphase project and was developed through cognitive interviews with hematopoietic cell transplantation caregivers, using verbal probing and think-aloud techniques [3,10,17-25].

### Sampling Frames

The sampling frames were email distribution lists from the National Bone Marrow Transplant Link (nBMTLINK) and Blood and Marrow Transplant Information Network (BMT InfoNet); both are nonprofit patient advocacy organizations in the United States devoted to serving transplant patients and family caregivers. With institutional review board approval, the nBMTLINK and BMT InfoNet advertised and provided access (ie, through hyperlinks) to the survey in their electronic newsletters and through email distribution lists. All listed members were presumed to have been sampled. Recruitment into the lists was voluntary and opt-in. Total counts of members in the lists and noncoverage of the target population were unknown. Additional survey responses were obtained by distributing a study brochure that contained the survey URL and QR code at BMT InfoNet’s Celebrating a Second Chance at Life Survivorship Symposium (May 2-5 2019, Orlando, Florida). A waiver of informed consent documentation was obtained, and information about the survey was provided on the first screen.

### Potential Error

Although there is no sampling error in a census (ie, all members of the email lists were sampled), there were other sources of potential error in surveys, such as nonresponse and measurement errors. The survey was implemented by the Center for Survey Research at Indiana University (LY); cognitive interview techniques [3] were used to minimize error in the development of the questionnaire.

### Data Collection

The survey was programmed for web administration in Qualtrics (Qualtrics XM) software. The field period was May 2 to June 30, 2019. Eligibility criteria included being an unpaid informal caregiver of an hematopoietic cell transplantation recipient, an adult, and able to complete the survey online in English. A US $20 gift card was offered to respondents for survey completion. The survey duration was approximately 16 minutes.

### Survey Components

The survey included 5 components: (1) caregiver characteristics (age, gender, race, ethnicity, marital status, educational status, employment, annual household income, relationship with care recipient, and caregiver medical conditions, for example, high blood pressure, heart disease, diabetes, arthritis, asthma, mental health disorder, cancer); (2) caregiving characteristics, responsibilities, and life experiences posttransplant (eg, care recipient’s age, gender, timing of transplant, transplant type, and transplant source, stem cell donor relationship, care duration, care burden, whether residing with the care recipient, whether caring for others in addition to the hematopoietic cell transplantation patient); (3) use of information technology, including the patient portal; (4) depression and anxiety; and (5) coping strategies [3]. For items 4 and 5, the Patient Health Questionnaire (PHQ-4), and Brief COPE were incorporated [26,27].

The PHQ-4 screens has depression and anxiety subscales consisting of 2 items each [26,28]. Respondents rate symptoms of depressed mood (eg, having little interest or pleasure in doing things) and anxiety (eg, not being able to stop or control worrying), over the past 2 weeks on a scale from 0 (not at all), to 3 (nearly every day). Subscale scores range from 0 to 6 with a cut-off score of 3, suggestive of clinically significant depressive or anxiety disorders, respectively. Higher scores indicate worse depression and anxiety, with Cronbach α=.85 when measured in a large general population sample [29].

Brief COPE is a 28-item instrument used to assess 14 different coping strategies: self-distraction, active coping, denial, alcohol and drug use, use of emotional support, use of instrumental support, behavioral disengagement, venting, positive reframing, planning, use of humor, acceptance, and religion [30]. The author provides permission to choose or adapt selected scales for use. Thus, based on cognitive interviews of hematopoietic cell transplantation caregivers, 16 items were included in the final survey [3]. Factor analysis yielded a set of 4 unique coping factors. The mean response to the component items in each factor served as each caregiver’s score for that factor.

### Statistical Analysis

We summarized continuous variables with means and standard deviations, and we summarized categorical variables with percentages. Logistic regression models were fit in 3 stages. First, we assessed the univariate and multivariate association of caregiver characteristics with use of the health care portal for the care recipient’s health. The multivariate model was determined by entering all variables at once and then removing one variable at a time (backward selection) until all remaining variables were statistically significant (ie, had odds ratios with 95% confidence intervals that excluded the value of 1.0). Second, we assessed univariate and multivariate associations of caregiver mental health measures with use of the health care portal for the care recipient’s health using the same approach. Third, we combined all the variables from the two multivariate models into a single combined multivariate model and further reduced variables with backward selection. These three modeling approaches were also repeated with the outcome changed to the caregiver’s use of a health care portal for their own health. The fit of all multivariate models was summarized by area under the curve (AUC), which ranges from 0.5 for a random model to 1.0 for a perfect model and quantifies how well the fitted logistic regression probabilities discriminate among caregivers who use the portal and caregivers who do not. Data were analyzed using R (version 3.6.02) in R Studio (version 1.2.5033).

## Results

### Caregiver Demographics

**Figure 1:**
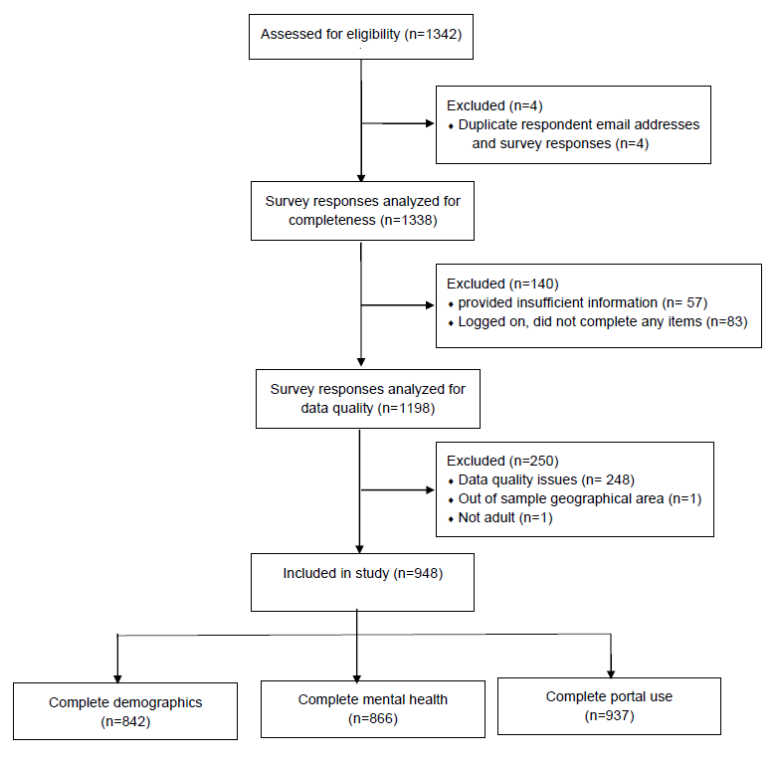
Flow diagram outlining number of eligible and responding participants to survey, as well as number of participants included in analysis.

A flow diagram of the survey respondents (N=948) is shown in Figure 1, and demographics are summarized in Table 1. Note that percentages are based on denominators that vary from the overall sample size of 948 due to missing data. The median age of the study population was 40 years (range 18-89 years). Most caregivers identified as female (620/944, 65.7%), were married (823/943, 87.3%), were employed (743/940, 79.0%), were White (746/940, 79.4%), were of non-Hispanic ethnicity (783/941, 83.2%), were college educated (665/945, 70.4%), and had annual household income greater than $50,000 (623/872, 71.4%). Caregiver relationships to care recipients were parent (311/946, 32.9%), adult child (274/946, 28.9%), spouse (257/946, 27.1%), and other (104/946, 11.1%; eg, grandparent, cousin, friend).

**Table 1:** Summary of caregiver demographics and caregiving characteristics.

Variables		Participants (excluding missing data)
**Age (years), n (%)**		
	≤40 years	479 (50.7)
	>40 years	465 (49.3)
**Gender, n (%)**		
	Male	324 (34.2)
	Female	620 (65.7)
**Income, n (%)**		
	≤$50,000	249 (28.5)
	$50,001-$99,999	373 (42.8)
	≥$100,000	250 (28.7)
**Race, n (%)**		
	White	746 (79.4)
	Other^a^	194 (20.6)
**Ethnicity, n (%)**		
	Hispanic	158 (16.8)
	Non-Hispanic	783 (83.2)
**Marital status, n (%)**		
	Married	823 (87.3)
	Unmarried	120 (12.7)
**Employment status, n (%)**		
	Employed	743 (79.0)
	Unemployed	197 (21.0)
**Education, n (%)**		
	Some college or less	280 (29.6)
	College degree or more	665 (70.4)
**Caregiver relation to recipient, n (%)**		
	Parent	311 (32.9)
	Child	274 (28.9)
	Spouse	257 (27.1)
	Other	104 (11.1)
**Donor relationship, n (%)**		
	Related donor	476 (51.0)
	Unrelated donor	328 (35.1)
	Patient themselves	130 (13.9)
**Caregiver supporting another individual, n (%)**		
	Yes	644 (68.1)
	No	301 (31.9)
**Care duration, n (%)**		
	≤6 months	443 (46.9)
	>6 months	501 (53.1)
**Care burden, n (%)**		
	≤20 hours/week	343 (36.4)
	20-40 hours/week	376 (39.9)
	>40 hours/week	224 (23.7)
**Caregiver lives with recipient, n (%)**		
	Yes	786 (83.4)
	No	156 (16.6)
Caregiver medical conditions, mean (SD)		1.2 (1.3)

^a^The race variable was a multiple choice question in our survey; however, since the majority of respondents were White, during analysis, we used only dummy code White/non-White.

### Caregiving Responsibilities and Characteristics

The majority of caregivers supported another individual in addition to the care recipient (644/945, 68.1%) and resided in the same household as the care recipient (786/942, 83.4%). Care demands varied from ≤20 hours per week (343/943, 36.4%), through 20 to 40 hours per week (376/943, 39.9%), to >40 hours per week (224/943, 23.7%). Duration of caregiving was almost evenly split between ≤6 months (443/944, 46.9%) and >6 months (501/944, 53.1%). Two-thirds of caregivers (629/948, 66.4%) indicated they had at least one chronic medical condition.

### Caregiver Mental Health

**Figure 2:**
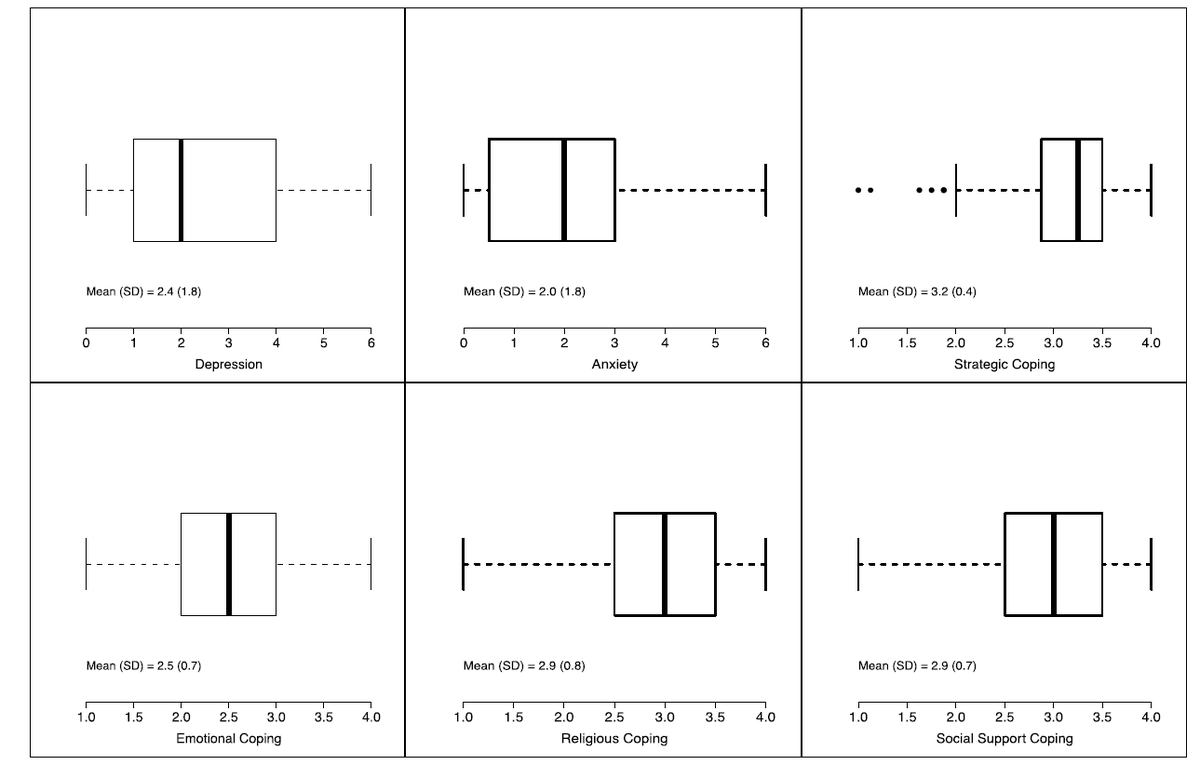
Summary of caregiver mental health characteristics.

Caregiver mental health variables are summarized in Figure 2; 28.6% of caregivers (259/904) exceeded the cut-off score of 3 for clinically significant depression, and 21.5% (194/903) exceeded the cut-off score of 3 for clinically significant anxiety. The means of the 4 coping scales ranged from 2.5 to 3.0, suggesting the 4 coping processes were used sometimes by the average caregiver.

### Care Recipient Demographics

Care recipient demographics are summarized in Table 2. Most (658/944, 69.7%) were adults and 63.3% (598/945) were male; 50.9% (476/934) received a transplant from a related donor, 35.1% (328/934) from an unrelated donor, and 13.9% (130/934) received an autologous transplant. Cell sources for the transplants varied among bone marrow (470/935, 50.3%), peripheral blood (113/935, 37.6%), and cord blood (113/935, 12.1%).

**Table 2:** Summary of care recipient characteristics.

Variables		Participants, n (%)
**Age (years) (n=944)**		
	<18 years	286 (30.3)
	≥18 years	658 (69.7)
**Gender (n=945)**		
	Male	598 (63.3)
	Female	347 (36.7)
**Timing of transplant (n=945)**		
	≤6 months	234 (24.8)
	7 months-1 year	197 (20.8)
	1-2 years	164 (17.3)
	2-3 years	188 (20.0)
	>3 years	162 (17.1)
**Transplant type (n=935)**		
	Bone marrow cells	470 (50.3)
	Cord blood cells	113 (12.1)
	Peripheral blood stem cells	352 (37.6)

### Health Care Portal Usage

Caregivers (597/937, 64%) accessed a health care portal for information regarding their care recipient’s health, 49% (463/937) accessed a health care portal for checking their own health information; 36.2% (340/937) accessed a health care portal for checking both (ie, self as well as care recipient’s), while 23.1% (217/937) did not access a portal for either purpose.

We report univariate correlations between demographics, mental health variables, and caregiver access of health care portals for their care recipients in Multimedia Appendix 1.

### Caregiver Factors Associated With Use of Care Recipient’s Health Care Portal

**Figure 3:**
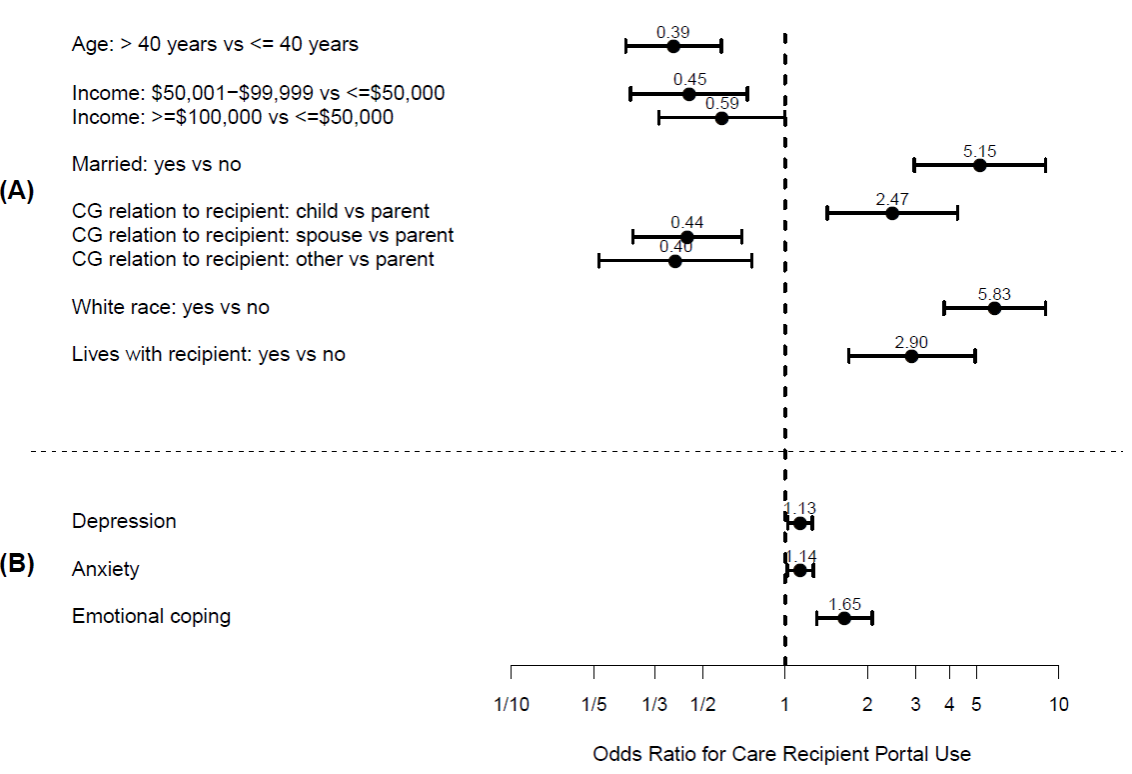
Multivariate odds ratios (dots) and 95% confidence intervals (bars) for (A) caregiver characteristics and (B) mental health for the use of a care recipient’s health portal. CG: caregiver.

**Figure 4:**
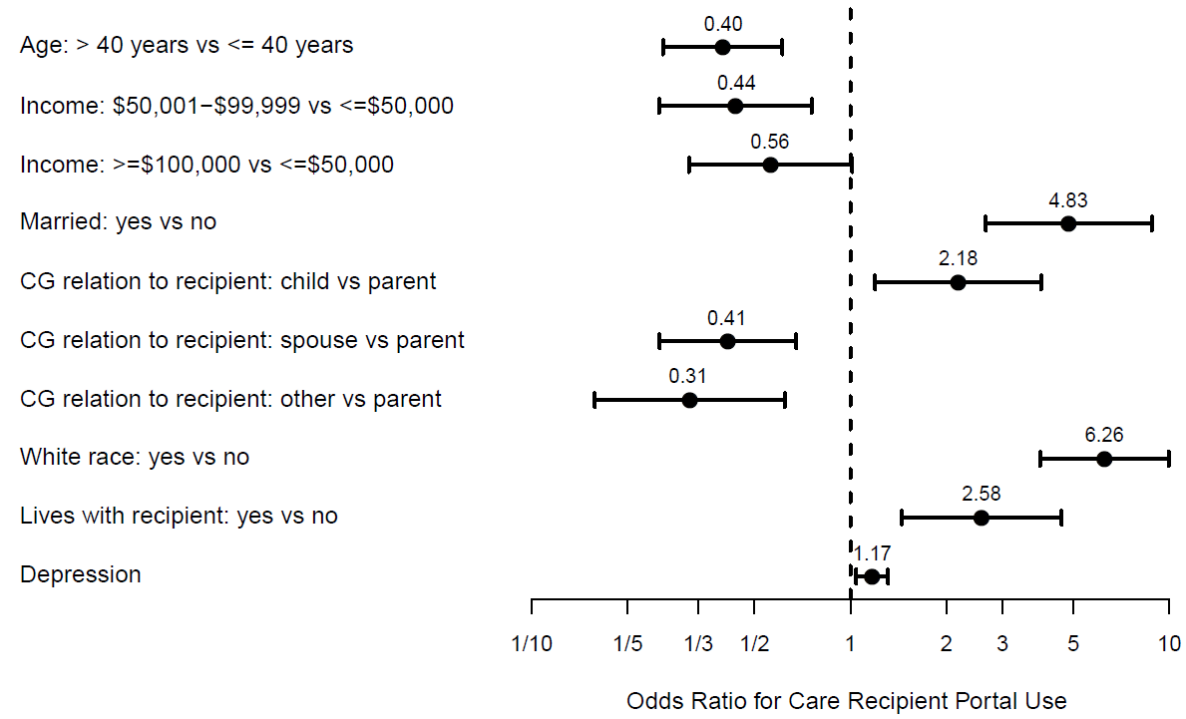
Multivariate odds ratios (dots) and 95% confidence intervals (bars) for combined caregiver characteristics and mental health for the use of a care recipient’s health portal.

In the multivariate model of caregiver demographics, care recipient portals were more likely to be accessed by White caregivers, 40 years old or younger, married, earning an income less than $50 000, caring for their parent, and living with their care recipient (Figure 3A; AUC 0.885). In the multivariate model of caregiver mental health variables, care recipients’ portals were more likely to be accessed by caregivers with higher depression, anxiety, and emotional coping (Figure 3B, AUC 0.668). However, in the final multivariate model that included both caregiver demographics and mental health variables, caregiver depression was the only mental health variable that remained associated with caregiver use of the care recipient portal while controlling for caregiver demographics (Figure 4; AUC 0.856).

### Caregiver Factors Associated With Use of Caregiver’s Health Care Portal

**Figure 5:**
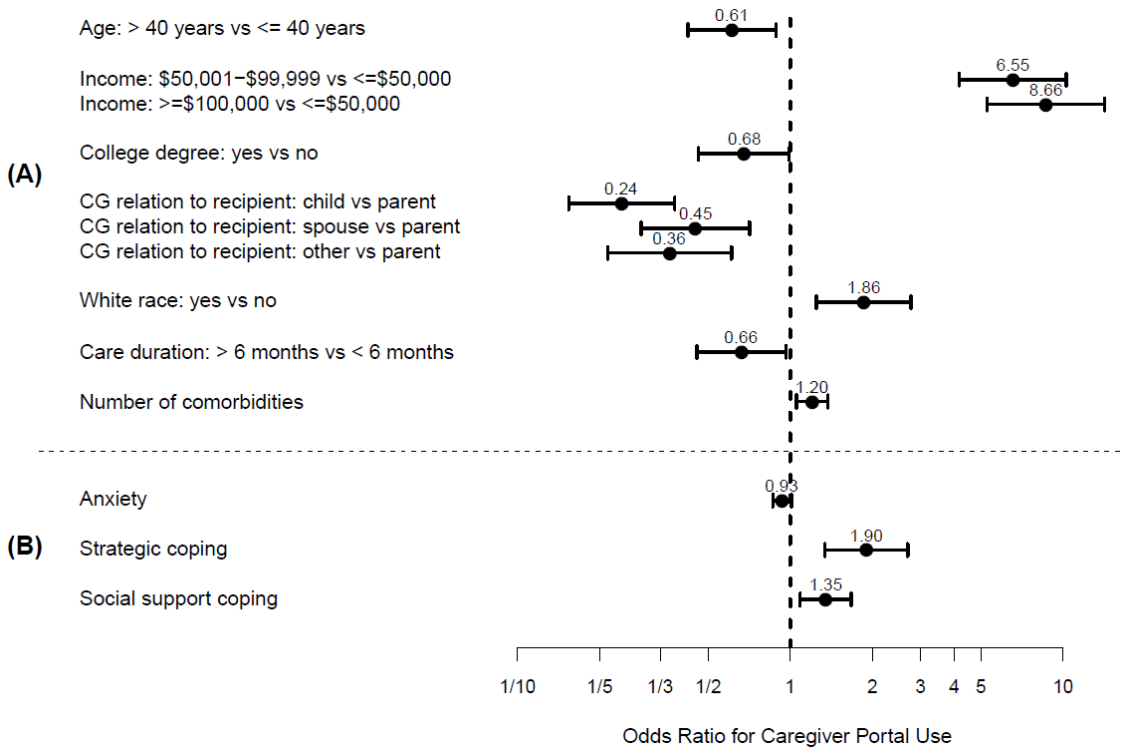
Multivariate odds ratios (dots) and 95% confidence intervals (bars) for (A) caregiver characteristics and (B) mental health for the use of a caregiver’s own health portal.

**Figure 6:**
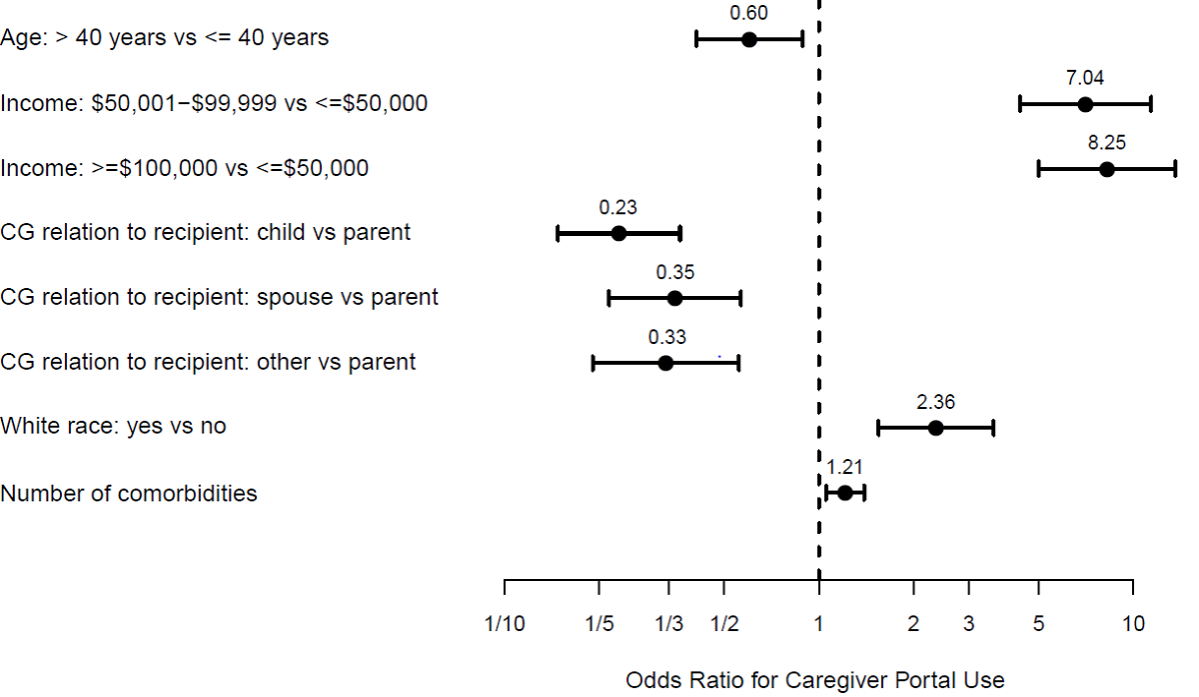
Multivariate odds ratios (dots) and 95% confidence intervals (bars) for combined caregiver characteristics and mental health for the use of a caregiver’s own health portal.

In the multivariate model of caregiver demographics, caregivers’ use of their own health care portal was more likely among White caregivers, age 40 years or younger, without a college degree, with high income (>$50,000), with care duration <6 months, and an increased number of medical comorbidities (Figure 5A; AUC 0.823). In the multivariate model of caregiver mental health variables, self-portal use was more likely with greater strategic and social support coping (Figure 5B; AUC 0.624). However, in the final multivariate model, lack of college degree, care duration, and strategic and social support coping were no longer associated with portal use (Figure 6; AUC 0.790) partially due to multicollinearity. Specifically, higher anxiety was correlated with shorter duration of caregiving, and increased use of social support coping was correlated with higher levels of education.

## Discussion

To our knowledge, this study of more than 900 caregivers from a national US sample is the largest published sample of hematopoietic cell transplantation caregivers surveyed to date focused on caregivers’ use of their own and their care recipients’ health portal [3,24,25,31]. Our study highlights hematopoietic cell transplantation caregiver demographics, mental health, coping behaviors, caregiving characteristics, and care recipient characteristics. We explored the relationship between caregiver characteristics, mental health and coping, and caregiver portal use for self as well as care recipient. Caregiver demographics—mostly female, married, White, employed, educated, and non-Hispanic—were consistent with those in a recently published single-institution, cross-sectional analysis of hematopoietic cell transplantation caregivers [32].

Caregivers in our sample experienced significant burden. Nearly two-thirds supported their care recipient for >20 hours a week and more than half supported their care recipient for over 6 months. These data support the findings of previously published studies [6,11,33] that have reported high levels of distress, depression, and anxiety in the hematopoietic cell transplantation caregiving population and the demands that caregivers must juggle across the hematopoietic cell transplantation trajectory. In addition, two-thirds of caregivers in our sample had at least 1 chronic condition, indicating additional challenges that may impact self-care or their own health.

The patient portal is expected to support patients and their families in managing their health and the health of their care recipient. In this sample, approximately two-thirds of caregivers accessed their care recipient’s portal, but nearly one-quarter of caregivers reported never accessing the portal for themselves or the care recipient. These estimates deviate from the findings of previous studies [13,14,34] that reported low caregiver access to the care recipient’s patient portal. Recent work in breast cancer suggests increased caregiver registration for the patient portal through a structured process of establishing a shared visit agenda and clarifying expectations about the role of family caregivers through a communication intervention, called Sharing in Care [35]. Such studies may allow us to examine strategies that are effective in supporting caregivers and engaging them in activities that may promote self-care as well as their care recipient. How self-care practices as well as quality of care provided by the caregiver influences subsequent patient outcomes remains a critical question in the field.

Our study provides insight into factors that may impact caregiver portal use of the care recipient. Being young, married, White, an adult child caregiver, and residing in the same household as the care recipient increased likelihood of caregiver portal use of the care recipient. These factors help identify where certain strategies could be targeted in future research (eg, older age, single, non-White, parent or other caregiver, separate living residences). It was encouraging that income was not a barrier to accessing the care recipient’s portal. Interestingly, caregivers who reported higher depression scores were also more likely to use the portal for their care recipient. Our group previously found that among users of a health information technology system (Roadmap 1.0), hematopoietic cell transplantation caregivers of adult care recipients who perceived Roadmap 1.0 to be more useful were those who reported lower quality of life and more fatigue, depression, and distress [23]. We speculated that caregivers who were struggling with the caregiving process may have consequently been more reliant on repeated viewing of the health information technology system to reaccess information that they may not have comprehended well or recalled effectively. Surprisingly, in the multivariate models, duration of caregiving, care burden in hours per week, and complexity of hematopoietic cell transplantation, indicated by type of hematopoietic cell transplantation, did not influence caregivers accessing their care recipient portal, despite the association on the univariate level. This suggests that the characteristics of caregivers themselves drive the care recipient portal use.

In addition to examining portal use for the care recipient, we were also interested in factors associated with self-portal use. We found that older hematopoietic cell transplantation caregivers, non-White, low income, adult children or spouses of care recipients, or those with chronic medical conditions may be at risk for not adopting self-portal use. Thus, an evidence-based understanding of the landscape of caregiving characteristics and portal use may allow us to effectively design and develop novel interventions systems (eg, mobile health apps, wearable sensors) that complement or integrate within existing patient portals and further enhance user operability. In this age of rapid technological advances, evolving use of health information technology (eg, telehealth), new therapeutic regimens, and increased demands placed on patients and families in the outpatient setting, it is an opportune and exciting time to develop health information technology systems that may support family caregivers and enhance their preparedness for the caregiving process—for themselves and for care recipients. Importantly, health care systems may need to develop structured processes to train patients and families in using technologies, such as self and care recipient portal use. Such interventions may have the potential to facilitate engagement with the patient portal among caregivers themselves, thereby enabling them to also support their care recipient.

Major strengths of this study include having a large well-characterized hematopoietic cell transplantation caregiver population derived from a national sample and contributing novel information about portal access by caregivers. The survey was developed with rigorous research methodology conducted in hematopoietic cell transplantation patients and caregivers, including think-aloud and verbal probing approaches [3,16,17]. Nonetheless, we recognize the limitations of the study, which include the cross-sectional design. The findings may not be generalizable across the trajectory of hematopoietic cell transplantation care. Although we attempted to control for time since transplant in our analyses, caregiver burden may be subject to changing challenges across different time points. Additionally, the respondents may inherently be less burdened, by having the time or energy to complete a survey (ie, care recipient is doing well posttransplant). Selection bias may have also been influenced by those who were adept at completing a web-based online survey. Importantly, while this caregiver population was from a national sample, the generalizability of the findings is limited to hematopoietic cell transplantation caregivers who were female, White, non-Hispanic, married, employed, high income, and educated. Finally, the survey was only conducted in English, which may have restricted non-English speaking, reading, or writing caregivers.

Our findings highlight the intensive burden placed on hematopoietic cell transplantation caregivers, impact of mental health, and coping strategies used. We anticipate that the findings will inform future research around caregiver use of and attitudes toward different types of technology (eg, wearables and mobile health). For instance, future studies could characterize hematopoietic cell transplantation caregivers’ use of these different types and reasons for engaging with such tools. Future work could also examine whether caregivers are likely to use a tool to help manage their own well-being and what such a tool would look like. While examination of caregiver use of other technology tools has been pursued in other contexts, little is known about use among hematopoietic cell transplantation caregivers. Understanding factors that support adoption of technology (eg, electronic health record portal use) will be critical in upcoming years as newer systems are developed and newer care delivery approaches are integrated in health care systems (eg, telehealth, telemedicine).

## Appendix

Multimedia Appendix 1 Univariate odds ratios (dots) and 95% confidence intervals (bars).

## Abbreviations

AUC: area under the curve
BMT InfoNet: Blood and Marrow Transplant Information Network
nBMTLINK: National Bone Marrow Transplant Link
PHQ: Patient Health Questionnaire

## References

[1] Copelan EA (2006). Hematopoietic stem-cell transplantation. N Engl J Med.

[2] Laudenslager ML, Simoneau TL, Kilbourn K, Natvig C, Philips S, Spradley J, Benitez P, McSweeney P, Mikulich-Gilbertson SK (2015). A randomized control trial of a psychosocial intervention for caregivers of allogeneic hematopoietic stem cell transplant patients: effects on distress. Bone Marrow Transplant.

[3] Kedroske J, Koblick S, Chaar D, Mazzoli A, O'Brien M, Yahng L, Vue R, Chappell G, Shin JY, Hanauer DA, Choi SW (2020). Development of a national caregiver health survey for hematopoietic stem cell transplant: qualitative study of cognitive interviews and verbal probing. JMIR Form Res.

[4] Gemmill R, Cooke L, Williams AC, Grant M (2011). Informal caregivers of hematopoietic cell transplant patients: a review and recommendations for interventions and research. Cancer Nurs.

[5] AARP, National Alliance on Caregiving (2020). Caregiving in the United States. AARP.

[6] Simoneau TL, Mikulich-Gilbertson SK, Natvig C, Kilbourn K, Spradley J, Grzywa-Cobb R, Philips S, McSweeney P, Laudenslager ML (2013). Elevated peri-transplant distress in caregivers of allogeneic blood or marrow transplant patients. Psychooncology.

[7] Applebaum AJ, Bevans M, Son T, Evans K, Hernandez M, Giralt S, DuHamel K (2016). A scoping review of caregiver burden during allogeneic HSCT: lessons learned and future directions. Bone Marrow Transplant.

[8] Bevans M, Sternberg EM (2012). Caregiving burden, stress, and health effects among family caregivers of adult cancer patients. JAMA.

[9] Applebaum FR, Forman SJ, Negrin RS, Blume KG (2004). Hematopoetic Cell Transplantation, fourth ed.

[10] Kaziunas E, Hanauer D, Ackerman M, Choi S (2016). Identifying unmet informational needs in the inpatient setting to increase patient and caregiver engagement in the context of pediatric hematopoietic stem cell transplantation. J Am Med Inform Assoc.

[11] Cooke L, Grant M, Eldredge DH, Maziarz RT, Nail LM (2011). Informal caregiving in hematopoietic blood and marrow transplant patients. Eur J Oncol Nurs.

[12] Williams LA (2007). Whatever it takes: informal caregiving dynamics in blood and marrow transplantation. Oncol Nurs Forum.

[13] Iott B, Raj M, Platt J, Anthony D (2020). Family caregiver access of online medical records: findings from the Health Information National Trends Survey. J Gen Intern Med.

[14] Wolff JL, Darer JD, Larsen KL (2016). Family caregivers and consumer health information technology. J Gen Intern Med.

[15] Wolff J, Berger A, Clarke D, Green J, Stametz R, Yule C, Darer J (2016). Patients, care partners, and shared access to the patient portal: online practices at an integrated health system. J Am Med Inform Assoc.

[16] Kedroske Jacob, Koblick Sarah, Chaar Dima, Mazzoli Amanda, O'Brien Maureen, Yahng Lilian, Vue Rebecca, Chappell Grant, Shin Ji Youn, Hanauer David A, Choi Sung Won (2020). Development of a national caregiver health survey for hematopoietic stem cell transplant: qualitative study of cognitive interviews and verbal probing. JMIR Form Res.

[17] Chaar D, Shin JY, Mazzoli A, Vue R, Kedroske J, Chappell G, Hanauer DA, Barton D, Hassett AL, Choi SW (2019). A mobile health app (Roadmap 2.0) for patients undergoing hematopoietic stem cell transplant: qualitative study on family caregivers' perspectives and design considerations. JMIR Mhealth Uhealth.

[18] Collins D (2003). Pretesting survey instruments: an overview of cognitive methods. Qual Life Res.

[19] Kaziunas E, Buyuktur A, Jones J, Choi S, Hanauer D, Ackerman M (2015). Transition and reflection in the use of health information: the case of pediatric bone marrow transplant caregivers.

[20] Maher M, Kaziunas E, Ackerman M, Derry H, Forringer R, Miller K, O'Reilly D, An LC, Tewari M, Hanauer DA, Choi SW (2016). User-centered design groups to engage patients and caregivers with a personalized health information technology tool. Biol Blood Marrow Transplant.

[21] Runaas L, Hanauer D, Maher M, Bischoff E, Fauer A, Hoang T, Munaco A, Sankaran R, Gupta R, Seyedsalehi S, Cohn A, An L, Tewari M, Choi SW (2017). BMT Roadmap: a user-centered design health information technology tool to promote patient-centered care in pediatric hematopoietic cell transplantation. Biol Blood Marrow Transplant.

[22] Runaas L, Hoodin F, Munaco A, Fauer A, Sankaran R, Churay T, Mohammed S, Seyedsalehi S, Chappell G, Carlozzi N, Fetters MD, Kentor R, McDiarmid L, Brookshire K, Warfield C, Byrd M, Kaziunas S, Maher M, Magenau J, An L, Cohn A, Hanauer DA, Choi SW (2018). Novel health information technology tool use by adult patients undergoing allogeneic hematopoietic cell transplantation: longitudinal quantitative and qualitative patient-reported outcomes. JCO Clin Cancer Inform.

[23] Fauer AJ, Hoodin F, Lalonde L, Errickson J, Runaas L, Churay T, Seyedsalehi S, Warfield C, Chappell G, Brookshire K, Chaar D, Shin JY, Byrd M, Magenau J, Hanauer DA, Choi SW (2019). Impact of a health information technology tool addressing information needs of caregivers of adult and pediatric hematopoietic stem cell transplantation patients. Support Care Cancer.

[24] Shin JY, Kang TI, Noll RB, Choi SW (2018). Supporting caregivers of patients with cancer: a summary of technology-mediated interventions and future directions. Am Soc Clin Oncol Educ Book.

[25] Shin Ji Youn, Choi Sung Won (2020). Online interventions geared toward increasing resilience and reducing distress in family caregivers. Curr Opin Support Palliat Care.

[26] Kroenke K, Spitzer RL, Williams JB, Löwe B (2009). An ultra-brief screening scale for anxiety and depression: the PHQ–4. Psychosomatics.

[27] Kershaw T, Northouse L, Kritpracha C, Schafenacker A, Mood D (2007). Coping strategies and quality of life in women with advanced breast cancer and their family caregivers. Psychology & Health.

[28] Spitzer RL, Kroenke K, Williams J B (1999). Validation and utility of a self-report version of PRIME-MD: the PHQ primary care study. Primary Care Evaluation of Mental Disorders. Patient Health Questionnaire. JAMA.

[29] Löwe Bernd, Wahl I, Rose M, Spitzer C, Glaesmer H, Wingenfeld K, Schneider A, Brähler Elmar (2010). A 4-item measure of depression and anxiety: validation and standardization of the Patient Health Questionnaire-4 (PHQ-4) in the general population. J Affect Disord.

[30] Carver CS (1997). You want to measure coping but your protocol's too long: consider the brief COPE. Int J Behav Med.

[31] Grossman MR, Zak DK, Zelinski EM (2018). Mobile apps for caregivers of older adults: quantitative content analysis. JMIR Mhealth Uhealth.

[32] Jamani K, Onstad LE, Bar M, Carpenter PA, Krakow EF, Salit RB, Flowers ME, Lee SJ (2018). Quality of life of caregivers of hematopoietic cell transplant recipients. Biol Blood Marrow Transplant.

[33] Von Ah D, Spath M, Nielsen A, Fife B (2016). The caregiver’s role across the bone marrow transplantation trajectory. Cancer Nursing.

[34] Wolff JL, Darer JD, Berger A, Clarke D, Green JA, Stametz RA, Delbanco T, Walker J (2017). Inviting patients and care partners to read doctors' notes: OpenNotes and shared access to electronic medical records. J Am Med Inform Assoc.

[35] Wolff JL, Aufill J, Echavarria D, Heughan J, Lee KT, Connolly RM, Fetting JH, Jelovac D, Papathakis K, Riley C, Stearns V, Thorner E, Zafman N, Levy HP, Dy SM, Wolff AC (2019). Sharing in care: engaging care partners in the care and communication of breast cancer patients. Breast Cancer Res Treat.

