# Supplementary figures and images for: Electronic Health Record Portal Use by Family Caregivers of Patients Undergoing Hematopoietic Cell Transplantation: United States National Survey Study
Source: JMIR Cancer. 2021 Mar 9;7(1):e26509. doi: 10.2196/26509 (PMC8086639; doi:10.2196/26509)

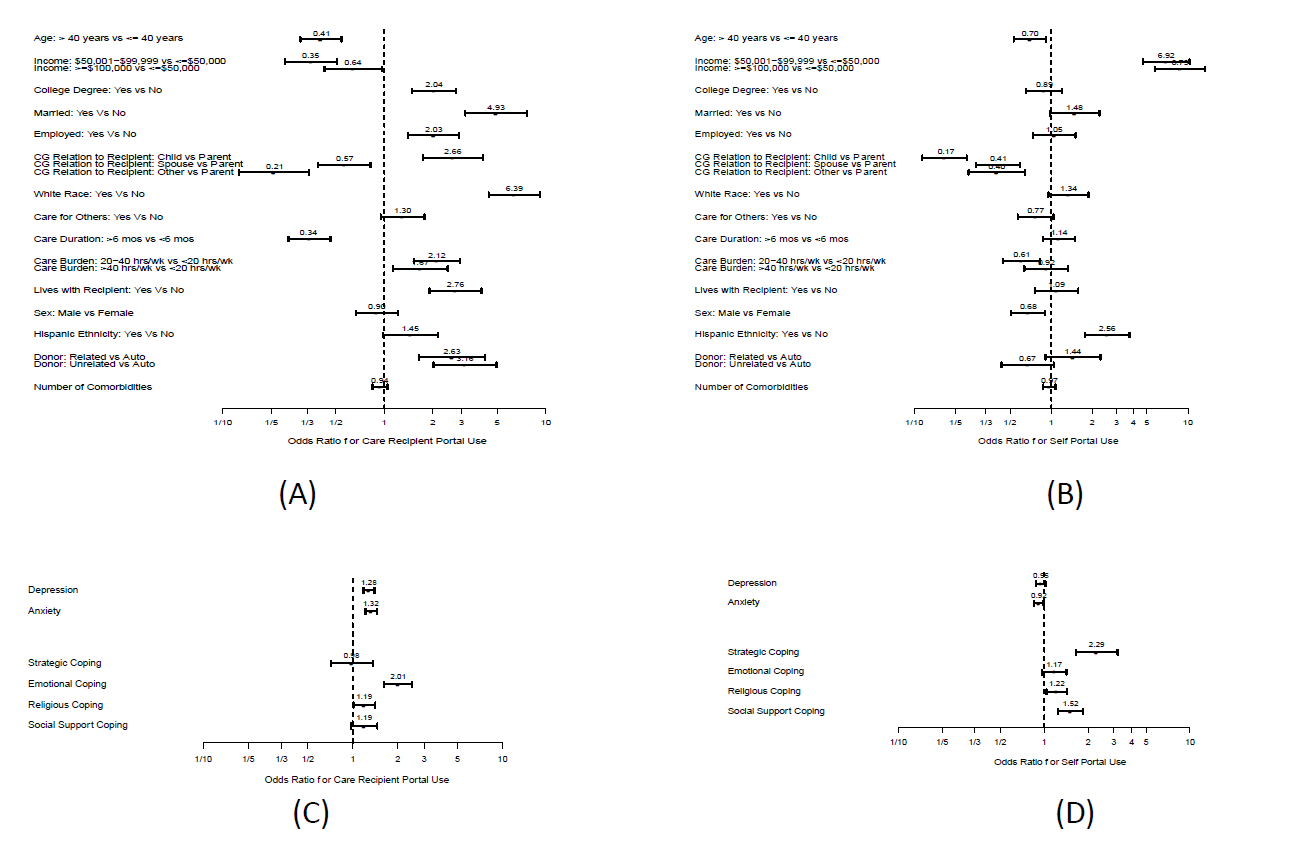

Supplement: Multimedia Appendix 1 [file cancer_v7i1e26509_app1.png]
